# Readiness to enter the workforce: perceptions of health professions students at a regional Australian university

**DOI:** 10.1186/s12909-022-03120-4

**Published:** 2022-02-09

**Authors:** Bunmi S. Malau-Aduli, Karina Jones, Faith Alele, Mary D. Adu, Aaron Drovandi, Gillian Knott, Louise Young, Clara Jo

**Affiliations:** College of Medicine and Dentistry, James Cook University, QLD 4811 Townsville, Queensland Australia

**Keywords:** Clinical practice, Student support, Training, Work-readiness, Mixed-methods

## Abstract

**Background:**

Perceived readiness for practice can help mitigate the stress and uncertainty associated with transitioning from university into the workforce. This study aimed to identify factors influencing the readiness for clinical practice among final-year medical, dental, and pharmacy students at an Australian regional university.

**Methods:**

The study utilised a sequential explanatory mixed-methods approach with surveys administered for the quantitative phase and interviews/focus groups for the qualitative phase. Descriptive statistics and inductive thematic analysis were utilised for the quantitative and qualitative data, respectively. Triangulation of findings from both phases facilitated in-depth understanding of the factors that influenced participants’ self-perceived readiness for clinical practice.

**Results:**

From the three disciplines, 132 students completed the survey and 14 participated in the focus groups and interviews. Students felt most prepared in their patient-centred capabilities, core skills, and advanced consultation skills, and least prepared in their system-related capabilities and clinical care skills. Themes identified as essential enablers and confidence builders in relation to workforce readiness in all three disciplines were: gained knowledge and skills, value of clinical placement experiences, support from peers, family and staff. However, students felt their work-readiness was impaired by heavy academic workloads and poor knowledge of health care systems, which affected skills development. Participants suggested additional support in health care system and clinical governance, mental healthcare, and induction to placement sites to further improve their work readiness.

**Conclusions:**

The findings of this study suggest that improving work-readiness of healthcare students requires alignment of learning needs to real-world practice opportunities, ensuring support systems are appropriate, and early familiarisation with the healthcare system.

## Background

Rapidly occurring global changes and best practice policies to foster innovation in education place pressure on higher education educators to make continuous adjustments to teaching in order to produce highly educated and skilled work-ready graduates [1]. Graduates are expected by employers to demonstrate essential workplace generic skills and competencies that foster smooth transition and integration into the workforce [2]. These essential workplace attributes include communication, teamwork, problem solving, resilience and commitment to life-long learning [3–6], which are indicative of graduates’ readiness for practice [7, 8].

Readiness for practice is an emerging field of educational research which focuses on graduating students’ work-readiness, and the identification and management of factors such as student familiarity with teaching curricula, assessment, placement expectations and an understanding of professional requirements after graduating, which influence transition into the workforce [8–11]. High levels of perceived readiness for practice can help mitigate the stress and uncertainty associated with transitioning from university into the workforce, particularly for new graduates in highly demanding and stressful work environments [8, 9] like the healthcare professions. The perception of readiness for practice is strongly linked to the quality of teaching, as well as individual-specific experiences and expectations [12, 13]. For healthcare students, this includes the utilisation of case-based learning, research and practical skills development, and inter-professional learning activities [14–16].

Students enrolled in health professional degrees may find the transition to workforce phase overwhelming, as a result of having to translate theoretical knowledge into clinical practice, with sudden significant contribution to and impact on patient health outcomes [17–19], and feelings of professional inadequacy and under-preparedness for clinical practice [12, 20]. This is compounded by the growing emphasis on lifelong learning, the expectation of undertaking ongoing self-directed career advancement activities [21] and drastically altered life after graduation [22]. Possessing appropriate support mechanisms is expected to facilitate positive learning outcomes and smoothen transition from university into the workforce. This is theorised to increase perceived work-readiness, including skills development and understanding of roles within the health system, and fostering of lifelong learning motivation and behaviours [8, 17, 23].

While a large body of research have explored entry-level work readiness of nursing students and interns [24–27], there is less research exploring the readiness for clinical practice among students in other health professions fields [4, 28–33]. Even fewer research has explored this topic across multiple health professions student groups within the same study [4, 34, 35]. Therefore, this study aimed to investigate perceptions of readiness for clinical practice and identify the key factors that influence work-readiness among final-year medical, dental and pharmacy students at a regional Australian university.

### Organisational Context and Objectives

James Cook University (JCU) is outcomes-focused from recruitment through to delivering qualified healthcare professionals for regional, rural and remote communities most in need. The College of Medicine and Dentistry (CMD) at JCU has a mandate to produce graduates capable of meeting the needs of North Queensland, Australia, with a unique focus on rural, remote, indigenous and tropical health. The CMD selectively recruits students from regional, rural and Indigenous communities or those who have an interest in ‘going rural’ into its health professions courses and provides students with diverse rural placement opportunities.

The CMD offers undergraduate training in medicine, dentistry and pharmacy. The first three years of the six-year medical program are predominantly pre-clinical, with introductions to the foundations of medicine, and some clinical skills sessions. The final three years are predominantly clinical, and involve tertiary, rural and remote hospital-based rotations, with the sixth year designated as the pre-internship year. The five-year dentistry program also delivers clinical exposure from year one through an observational placement. In year two, students participate in community placements and clinical experiences at the JCU Dental clinic. In years three and four, students deliver dental care for patients at JCU Dental under the supervision of experienced dental professionals. In year five, students develop their clinical skills through semester-long clinical placements with public dental health services across regional, rural and remote locations in Queensland, Tasmania and the Northern Territory. Similarly, the four-year pharmacy program delivers a pre-professional curriculum in years one and two and an integrated professional curriculum in years three and four. Brief exposure to clinical experiences occurs in years one and two. In third and fourth year, students undertake placements in community and hospital pharmacies in urban, regional, and rural locations.

## Methods

This research employed a sequential, explanatory mixed-methods approach, which included the collection and analysis of both quantitative data (through hard copy and online surveys), and qualitative data (through interviews and focus groups discussions [FGD]) from medical, dental, and pharmacy students at JCU, a regional university in North-East Australia. Surveys were administered between August and October 2019, and interviews/FGD were conducted in October and November 2019. The JCU Human Ethics Review Committee granted ethics approval for the study (H7853).

### Participants

All final year students in the medicine (185 students), dentistry (68 students) and pharmacy (56 students) programs were invited to participate in the study. Students were recruited through an announcement during one of their scheduled class times one week before the scheduled survey date. Students were assured of no adverse academic repercussions for non-participation and data collection was conducted by the research team members (BMA and AD) who were not involved in the teaching of these student groups. Furthermore, as an incentive, participants were entered into a draw to win one of three available $50AUD gift cards.

### Surveys

Surveys were adapted with permission from two validated tools [8, 17]. Each survey assessed student perceptions of their clinical training experiences, focusing on six skill clusters: core skills, advanced consultation skills, personal and professional capabilities, patient-centered capabilities, clinical care, and system-related capabilities. Demographic questions were identical for all three disciplines (including age, gender, origin, hometown rurality, family members being university educated or health professionals, and previous work in a health field). These were followed by 5-point Likert-scale statements, rated from 1 (not prepared) to 5 (extremely prepared). Medical students had 45 statements, dental students had 49, and pharmacy had 40 (see Table 1) with 36 of these statements being identical (or similarly worded) between the three disciplines. The remainder were discipline-specific to reflect skills and attributes relevant to each discipline. Participants also commented on three open-ended questions relating to: (1) factors that have been beneficial for work readiness, (2) the most challenging aspects of getting ready for the workforce, and (3) additional support measures that could be provided to students in preparation for their transition.

**Table 1:** Student ratings of Likert-scale statements, segmented by cluster

Statement	Scores Mean (SD)	% WP	% PP	% NP
**Cluster 1: Patient-Centred Capabilities**	**4.18 (0.54)**			
Understanding the concept of patient-centred practice	4.35 (0.69)	88.2	11.8	0
Overall patient-centred practice and humane care	4.25 (0.73)	87.1	10.8	2.2
Understanding the impact of patient-centred practice	4.24 (0.68)	88.2	10.8	1.1
Providing appropriate care for people of different cultures	4.19 (0.73)	83	16	1.1
Recognising the social and emotional factors in illness and treatment	4.14 (0.84)	77.7	19.1	3.2
Exploration of patient needs	4.12 (0.72)	79.6	20.4	0
Shared decision-making management	4.11 (0.70)	80.6	19.4	0
Understanding the relationship between primary/social and hospital care	4.03 (0.81)	75.5	21.3	3.2
**Cluster 2: Advanced Consultation Skills**	**4.14 (0.63)**			
Understanding health literacy and its impact on informed consent	4.26 (0.72)	84.1	15.9	0
Educating patients (health promotion and public health)	4.23 (0.80)	79.8	19.1	1.1
Communicating effectively and sensitively with patients and relatives	4.21 (0.79)	79.8	19.1	1.1
Being comfortable with the craft of consultation	3.87 (0.78)	69.1	27.7	3.2
**Cluster 3: Core Skills**	**4.09 (0.58)**			
Taking a history	4.31 (0.67)	90.4	8.5	1.1
Understanding my scope of practice and when to refer	4.25 (0.79)	84.1	13	2.9
Examining patients	4.23 (0.66)	90	8.8	1.3
Skills of close observation	4.09 (0.77)	77.2	21.5	1.3
Selecting appropriate investigations and interpreting the results	3.99 (0.79)	76.3	20	3.8
Clinical reasoning and making a diagnosis	3.95 (0.80)	72.3	24.5	3.2
Prescribing/dispensing safely	3.78 (0.99)	66	23.4	10.6
**Cluster 4: Personal and Professional Capabilities**	**3.85 (0.56)**			
Acting in a professional manner (with honesty and probity)	4.36 (0.82)	85.1	11.7	3.2
Working effectively in a team	4.19 (0.77)	78.7	21.3	0
Being aware of your limitations	4.15 (0.75)	83.8	13.8	2.5
Communicating effectively with colleagues	4.11 (0.80)	77.7	20.2	2.1
Engaging in self-critique of practice and clinical encounters	4.09 (0.71)	78.7	21.3	0
Engaging in self-directed lifelong learning	4.00 (0.83)	73.4	24.5	2.1
Coping with ethical and legal issues (such as confidentiality and consent)	3.95 (1.01)	72.3	18.1	9.6
Ability to cope in a resource-poor clinical setting	3.77 (0.94)	63.8	27.5	8.7
Understanding the purpose and practice of appraisal	3.72 (0.91)	60.6	29.8	9.6
Role modelling to junior colleagues	3.71 (0.88)	58.5	34	7.4
Managing your health, including stress	3.51 (1.00)	52.1	31.9	16
Coping with uncertainty	3.47 (0.88)	45.7	43.6	10.6
Undertaking a teaching role	3.06 (1.04)	31.6	38.3	29.8
**Cluster 5: Clinical Care**	**3.76 (0.86)**			
Maintaining good quality care	4.14 (0.72)	79.8	20.2	0
Using evidence and guidelines for patient care	4.14 (0.77)	80.9	17	2.1
Early management of emergency patients	3.74 (0.91)	66.3	23.8	10
Basic nutritional care (M, P)	3.03 (1.03)	31.6	36.8	31.6
**Cluster 6: System-related Capabilities**	**3.70 (0.60)**			
Ensuring and promoting patient safety	4.26 (0.69)	86.2	13.8	0
Reducing the risk of cross-infection	4.17 (0.79)	81.9	17	1.1
Keeping an accurate and relevant medical record	4.12 (0.83)	78.7	19.1	2.1
Time management	3.70 (0.83)	59.6	34	6.4
Organisational decision making	3.68 (0.91)	62.8	26.6	10.6
Clinical governance	3.47 (0.86)	48.9	39.4	11.7
Reporting and dealing with error and safety incidents	3.45 (1.04)	54.3	26.6	19.1
Using informatics as a tool in medical practice	3.41 (0.99)	46.8	34	19.1
Using audit to improve patient care	3.06 (1.11)	31.9	39.4	28.7

% WP (well prepared): those who responded with ‘Well-prepared’ or ‘Extremely prepared’

% PP (prepared): those who responded with ‘Prepared’

% NP (not prepared): those who responded with ‘Not prepared’ or ‘not very well prepared’

### Focus group and interview discussions

To recruit participants for the FGD and interview, the last question in the survey sought participants’ interest in providing further explanation about their preparedness for practice. Interest was indicated by providing their phone number and a suitable contact time, through which participants were invited to the FGD or interview session. A FGD (dental students) and telephone interviews (medicine and pharmacy students) were conducted two months after the survey to build on experiences and perceptions of readiness for practice that were identified in the surveys. The timing allowed for reflection as students were close to completion of their degree. To ensure that students were not coerced into participation or coerced into giving positive responses, research team members (BMA and AD) who were not involved in the teaching of the participants administered the focus groups and interviews. The authors have prior methodological experiences in conducting qualitative research. To encourage the provision of rich and detailed data by participants, emphasis was placed on honesty, anonymity and respondent confidentiality. The interview/discussion guide was developed by the research team to generate information on factors that influenced readiness for practice and possible recommendations. The interview guide was pilot tested before final use. Discussions were audio-recorded, and lasted 20–30 min for telephone interviews, and 60 min for the focus group, which took place in an informal classroom setting.

### Data analysis

Descriptive statistics, including mean scores and frequencies of participants’ sociodemographic characteristics and their survey responses were calculated in SPSS vs23. Qualitative data analysis was conducted in line with the consolidated criteria for reporting qualitative research (COREQ) [36]. Data from the open-ended survey questions, interviews and FGDs discussions were transcribed verbatim and assigned a code. Given that participants were busy preparing for their final exams, we were unable to return transcripts to participants for member checking. However, shared understanding of the responses provided was facilitated through summarizing interview/FGD accounts with participants at the end of each session. Repeat FGD or interview were not required. Transcripts were independently coded and analysed by two authors (BMA and AD) using inductive thematic analysis [37]. The analytical process included an initial line-by-line open coding of the content of the transcripts, followed by further refining and grouping of the codes into themes and subthemes using a constant comparison process, as advocated by Corbin and Strauss [38]. This process was repeated until no further themes were identified and data saturation was reached [39]. Discrepancies were resolved through consensus meeting to enhance the credibility of the findings. Triangulation of findings allowed identification of points of convergence and divergence from both the quantitative and qualitative phases of the study. Results from both phases of the study were integrated to provide further explanations and interpretations of the overall findings. The triangulation was also used to increase research rigour, validity and trustworthiness of the data [37]. Themes were presented using illustrative quotes affixed with participant age, gender (M/F), and degree pathway (Medicine [Med], Dentistry [Dent], and Pharmacy [Pharm].

## Results

### Quantitative:

Of the students invited to participate (n = 309), 132 (43%) completed the survey; (55/185 [30%] in medicine, 56/68 [82%] in dentistry, and 21/56 [38%] in pharmacy). Participants’ ages ranged from 20 to 39 years. The characteristics of the participants are summarised in Table 2. The participants were mostly domestic female students, with a mean age of 24 years, mostly from a major city or regional centre. Majority of the participants were not the first in their family to attend university but had a family member with experience as a health professional.

**Table 2:** Characteristics of the sample population (n = 132)

Characteristics	Numbers	Percentage
Participant Gender		
Male	51	38.6
Female	81	61.4
Student Origin		
Domestic	109	82.6
International	23	17.4
Hometown Rurality		
Major city	49	37.1
Regional centre	48	36.4
Rural town	33	25.0
Remote community	2	1.50
First in Family		
Yes (immediate)	36	27.3
No	96	72.7
Family Health Professional		
Yes	73	55.7
No	58	44.3
Prior Education		
Yes	32	24.2
No	100	75.8
Prior Health Experience		
Yes	15	11.4
No	117	88.6

### Ratings of Likert-scale statements:

Table 1 outlines the percentage response for each statement in the six skill clusters (Core skills; Advanced consultation skills; Personal and professional capabilities; Patient-centred capabilities; Clinical care and Systems-related capabilities) assessing opinions about readiness for practice. The participants’ mean scores were highest for patient-centered capabilities (4.18 ± 0.54), advanced consultation skills (4.14 ± 0.63) and core skills (4.09 ± 0.58). Scores in the areas of personal and professional capabilities (3.85 ± 0.56), clinical care (3.76 ± 0.86) and system related capabilities (3.70 ± 0.60) received lower ratings.

In relation to patient-centered capabilities (Cluster 1), majority of the participants felt they understood the concept and impact of patient-centered practice (88.2%), providing multicultural care (83.0%) and shared decision-making (80.6%). Responses on Advanced consultation skills (Cluster 2) highlighted that the participants understood the importance of health literacy and its impact on informed consent (84.1%), communicating effectively (79.8%) and educating patients (79.8%). With respect to core skills (Cluster 3), participants felt they were well prepared for history taking (90.4%), examining patients (90%) and understanding their scope of practice (84.1%). However, they felt less confident in their clinical reasoning (72.3%) and ability to prescribe or dispense safely (66.0%).

In relation to Personal and professional capabilities (Cluster 4), only 32% and 46% of participants felt prepared for undertaking a teaching role and coping with uncertainty respectively. For clinical care (Cluster 5), majority of the participants were well prepared to use evidence and guidelines for patient care (80.9%) and maintain good quality care (79.8%). However, participants felt least prepared for providing basic nutritional care (31.6%). In the system related capabilities (Cluster 6), participants were well prepared to ensure and promote patient safety (86.2%) and reduce cross infection (81.9%). Though, less than half of the participants felt prepared for clinical governance (48.9%) and the use of informatics as a tool (46.8%), while only one-third of the participants were prepared to use audit to improve patient care (31.9%).

### *Qualitative*:

The qualitative phase of the study included all survey participants' responses to the open-ended questions as well as the interviews/FGD. Initially, 28 students indicated interest in the interviews. However, only 14 respondents (11 in FGD and 3 in the interviews) participated. The low numbers could have been due to the late timing of the interviews as majority of the participants had completed their final exams and moved on.

#### Enablers of Work Readiness

Three major themes were identified as enablers and confidence-builders in relation to workforce readiness. These were knowledge and skills gained, value of clinical placement experiences as well as support from peers, family and academic staff.

### Theme 1: Knowledge and skills gained

Overall, the students felt their respective training programs had prepared them adequately to enter into the workforce. Theoretical knowledge, interpersonal communication and clinical skills were identified as important attributes for a smooth transition into the health workforce. The participants felt these attributes had helped them gain confidence and competence for the work ahead of them.

*I feel the medical school has done a very good job preparing its students for work life. I think it’s great that we get clinical experience from the first year and I think it’s great that it builds up over the years as well* (23, F, Med).

*I feel the onus is on the individual. I don’t believe there is a great deal a university degree can do apart from provide education and give placement opportunities, which JCU pharmacy does brilliantly already* (24, M, Pharm).

*In the clinical years, it’s been very good. I’ve seen a wide array of things and got to do a lot, which is really good. Having the clinical knowledge that I have; communication, practiced experience and patient management skills* (22, M, Dent).

### Theme 2: Value of clinical placement experiences

Students in all three disciplines identified placement as a key contributing factor to their knowledge and skills development in transitioning into the healthcare workforce. This perceived value of the placement experience was attributed to a combination of factors, most notably placement duration and alignment of the placement experience to real-world practice, which fostered continuous exposure to the workforce and enhanced the students’ skills acquisition.

*I absolutely feel ready to do basic dentistry as a general practitioner graduate. So, I can’t imagine not having this clinical placement year, I think, 90% of what I have learnt in clinic dentistry was from this year. And the placement opportunities was fantastic.* (28, F, Dent)

*A lot of 6th year placement is useful because it allows me to work as an ‘intern’ but with a little less responsibility and paperwork* (23, F, Med); *Continuous exposure to the workforce through placements were by far the most impactful in helping transition to the workforce* (23, M, Med).

*Completing summer placements and forming networks helped with job preparation* (24, F, Pharm).

It also included undertaking placements in rural, remote, and overseas settings, which provided practical experiences and learning opportunities that are unobtainable in urban settings.

*Being able to experience challenges faced by many remote communities with limited access to dental care* (23, M, Dent).

*I believe the rural placements prepare us very well for the transition to the workforce* (23, F, Med).

Supervisor support was a key contributor to the placement experience, with close supervision and structured clinical activities increasing the quality of the placement experience.

*Close supervision particularly on diagnosis and treatment planning helped increase my confidence* (22, F, Dent).

*Having that sort of emotional support from supervisors and then reassuring you…I think that makes you a bit more confident* (23, F, Dent).

However, some students felt they had limited supervisor availability at their placement, which negatively impacted the quality of the experience for them.

*I felt quite thrown into the deep-end because it was my first day and I was in the clinics and I just got sort of left [on my own]and they said ‘see your own patients’* (25, F, Med).

### Theme 3: Support from friends, family and academic staff

The participants emphasised the value of having friends and peers to rely on for support. Having friends who were recent healthcare graduates, or parents or other family members with medical or dental degrees was also seen as being indispensable. Family members were able to help in improving students’ understanding of teaching materials, completing assessments, developing clinical skills, and for general reassurance and support.

*Support from peers in the exact same position. There is a camaraderie that helps you adapt and push forward* (23, M, Med).

*I have a very understanding and supportive partner, friends and family who have helped guide me through applications and uncertainties*. *Support from my friends and hearing stories from other health practitioners about how they overcame their problems in the workforce helped my transition* (23, F, Dent).

*Having a parent who is currently in the health workforce was an asset as they are able to support me and give advice about life in the hospital. I feel I am able to reach out to this person for advice and tips. I also had help from multiple friends who are working as junior doctors that gave insight into what practicing is like in the hospital environment, which helps to somewhat mentally prepare myself* (23, F, Med).

Pharmacy students more often relied on individualised support from lecturers, which was possible due to the smaller class size in the discipline. Students felt support received from staff helped them to apply their knowledge in real-world situations and to work on their perceived weaknesses.

*Support from the staff has also been incredibly useful, whether it be for academic purposes or even having a casual conversation* (21, M, Pharm).

*The small class sizes allow staff to identify students’ strengths and weaknesses. In doing so, the staff supported students in helping them gain the confidence to improve upon their weaknesses prior to entering the workforce. This was especially useful in the counselling and dispensing practicals* (27, M, Pharm).

#### Barriers to Work Readiness

Whilst students generally felt their clinical knowledge and skills were adequate for practice, two key themes emerged as impairments to the participants’ confidence in their work readiness: (i) heavy academic workloads and (ii) poor knowledge of the health system and clinical governance structure.

### Theme 1: Heavy academic workload

Students’ academic workload particularly while on placement was perceived as a barrier to learning key clinical and technical skills, which created uncertainty in transitioning into the workforce as practice-ready health professionals.

*The draining hours of unpaid community placement as a final hurdle prior to commencing work as a health professional [was challenging]* (22, M, Pharm).

*Year 5 is obviously a lot of work. It’s the full year of clinical attachment and then you have to do your lecture work in your own time as well. I have to go home and leave my clinical attachment to study because I need to pass my exams* (24, M, Med).

*I don’t know if it was difficult, but it was just like a learning process, going from seeing not many patients, to seeing 8–12 patients and then, time management, remaining stress free, even though the last patient caused you stress, or even if you cause yourself stress, and then you’ve got to try and remain calm and fresh for the next patient* (22, F, Dent).

### Theme 2: Poor knowledge of the health system and clinical governance structure

Participants felt unprepared for the administrative and clinical governance aspects of the healthcare system. Confidence in areas of practice such as time management, meeting business targets and expectations of being an intern were perceived as lacking within all the three degree pathways, which is evidenced by the lower ratings in the ‘system-related capabilities’ cluster in the survey.

*I think what I’m most unprepared for in the workplace is the change in setting from a large dental school, where I’m still a student to being a dentist in January. It’s going to be a completely different set-up within the team - what is expected of me, who answers to me, and who I answer to, the whole dynamics is going to change, and I think that’s what I’m most unprepared for. The social interaction and the social side of dentistry and even the business side of dentistry, that’s something the dental school doesn’t like to talk about. They teach us the practice of dentistry, the medicine of dentistry. Unfortunately, a lot of us are going to go into private practice and that is the business of dentistry and, that is what scares me the most, having to talk about the money, having the hard conversations with a practice manager about not meeting targets*. (23, F, Dent)

*Well, the staff…they definitely had their expectations of me. Everyone behaves a little differently…for me I just have to take a step back and think ‘what do they really want from me?’ and when I did that, I found that I was much better with the team* (22, M, Pharm).

#### Recommendations to Further Improve Preparedness for Practice

Participants gave some recommendations, which could foster readiness for practice. These were in the areas of improved support for mental health care and building of resilience/adaption skills that were perceived as currently lacking in their curricula.

*Perhaps more work on mental health services and teaching us ways to build resilience* (23, F, Med).

*More resources on adapting to difficult work situations and environments in pharmacy practice* (27, M, Pharm).

*More clinical scenarios and model answers in clinical years to cover cases that we may not encounter clinically* (23, F, Dent).

In addition, students advised that the faculty could provide improved training on health system and clinical governance structure. They noted that improved preparation in the areas of professional registration, job interviews and resume preparation, meeting business targets and expectations of being an intern could enhance their confidence in being work ready. Participants wanted “*more information on the registration process itself after completing exams*”, “*speciality specific career assistance*” and “*practice interviews*” with “*sessions focusing on what is expected of interns, potentially going through some of the paperwork that [interns] might be expected to do*”.

*I think it would be beneficial for students if the College began discussing career paths within medicine from an earlier time in the degree* (25, F, Med).

*It was stressful knowing that students from other universities had started applying and looking into job applications at the start of the year whereas we started later* (23, F, Pharm).

Recommendations for placement improvement included better induction into placement sites, management of student expectations, and more standardised marking of assessment pieces between supervisors and across placement sites, to provide clarity for the supervising teams. Many students believed that supervisors had a ‘central tendency’ marking attitude, rarely giving out higher grades, which created uncertainty in their skill proficiency and some distrust in the grading system.

*Ensure supervision in each placement are equally well supported, with standardised training and checklists* (22, F, Dent).

## Discussion

This study investigated factors influencing health professions students’ readiness for clinical practice. The uniqueness of the study is linked its cross-professional relevance because it explored perceptions of work-readiness among students across multiple health professions who were trained with the focus on rural, remote, Indigenous, and tropical health. The study findings provide rich understanding of the participants’ learning experiences and perceptions of readiness for clinical practice to meet the healthcare needs of underserved communities. Furthermore, the study revealed gaps in competency-based elements required to prepare graduates for system-related clinical governance within the healthcare system.

Congruent with previous studies, the participants perceived that they were adequately prepared for practice in terms of patient-centred capabilities, core skills, and advanced consultation skills, particularly in relation to clinical/practical skills, interpersonal and communication skills, and teamwork [8, 30, 40]. These positive findings could be attributed in part, to the gains from clinical placement, which was a key avenue to pre-graduation exposure to the clinical environment, as indicated in the interviews. Students noted that through increased exposure to clinical practice, they had improved confidence in professional skills, such as taking medical histories and providing patient-centred care. Enhancement of students’ understanding of clinical practice, professional knowledge and practice through clinical placement experience have been previously reported [41]. Therefore, the current study confirms the pivotal role of clinical placements in students’ development of essential clinical skills for effective partnering with patients and their families for improved health outcomes.

The rural focus of the JCU program and related placement experiences was also seen as important in skills development, providing care for people from different cultures, coping in a resource-poor clinical setting, and being socially accountable. A systematic review of rural dental placement experiences found that rural placements enhanced basic clinical skills as well as clinical confidence [42]. A study of Australian dental graduates reported that students who have attended rurally focused universities were more likely to work in regional and rural locations [43], with similar findings amongst general practitioners who underwent training in remote Australian settings [44]. Such findings are vital for maintaining a rural health workforce, which has been an ongoing issue in ‘outback’ Australia [45]. These rural placement experiences may be similarly improved and result in a stronger rural workforce through positive role-modelling from supervisors, a supportive learning environment, familiarity with rural hospitals, and faculty members with significant rural experience [46–48].

Similar to previous studies [8, 32], the practice areas of system-related capabilities and clinical care skills, particularly in undertaking a teaching role, basic nutritional care, clinical governance and using audit to improve patient care were capabilities that received the least ratings from participants. Theses findings indicate perceived shortcomings in students’ training and clear opportunities to further improve undergraduate medical, pharmacy and dental education, to ensure that graduates feel well-prepared at the commencement of practice. Providing focused educational support in particular elements of these practice areas could further improve perception of preparedness and ability to cope with tasks and roles in real world situations.

This study identified the notable role of ‘external influencers’ in students’ self-perceived work readiness, including family and friends with healthcare experience. Studies have previously demonstrated that friends and family with experience in a relevant health profession are vital in obtaining skills for this transitional phase [49, 50]. Furthermore, as also noted in this study, the role of supervisors and their impact on the placement experience have been highlighted in previous studies with allied health students, medical, dental, and pharmacy students [51], and postgraduate medical trainees [52]. Supervisor’s style or nature of supervision, leadership skills and the extent of interaction with students are critical to foster positive learning experience during clinical placement [41]. Strategies to enhance supervisory style include the use of humour, enthusiasm and empathy, which could facilitate collaborative learning and improve students’ integration into their clinical role [53].

Despite being delivered by the College, non-academic teaching materials and skills such as resume preparation, job interviews, career guidance, and professional registration were seen as lacking or insufficient. Furthermore, it was emphasised that the administrative roles and the technical aspects of clinical practice needed more discussion to aid preparation for work. These have been found in previous research to be essential for students’ self-perceived readiness to practice [13, 32]. An approach that provides opportunity to enhance these skills is work integrated learning (WIL) as reported in other professions, such as business, industry, community organisations and social work [54]. WIL is recognised as making significant contribution to the readiness of graduates for the workplace, with provision of generic skills such as teamwork, organisational skills and communication. These are in addition to improved personal attributes such as confidence, resilience and discipline [54]. Medicine was one of the original WIL settings but has focussed mainly on clinical education, with lesser focus on the development of personal attributes [54]. On the other hand, dentistry and pharmacy have embedded career development into their final year capstone programs. However, in this study, all student groups have expressed a desire for further support in this area. In light of these findings, considerations should be given to expanding the content learnt in these topic areas.

Suggestions on familiarity with placement sites are also potential improvement opportunities on placement experiences. Familiarity with placement sites may be fostered in the early years [48]. Furthermore, the experiences can be enhanced through improved standardization of expectations, reduced workload, and enhanced supervision while on placement. Johnson et al., [55] found that experienced and supportive clinical supervisors, who provided mentorship, consistent training and good role-modelling, were fundamental to the success of a rural dental clinical placement program. However, practising health professionals are often time poor, and the delivery of such experiences may conflict with their pre-existing responsibilities. Nonetheless, this may be mitigated through appropriate training, support, and resources as these have been shown to ensure quality and consistency for placement activities [56].

Overall, the three groups of health professions students in this study emphasised the importance of high-quality placement experiences, enriched by skills acquisition and the availability of effective support systems (especially by supervisors, family and peers), to enhance work-readiness and ultimately patient outcomes. These activities support students’ self-perceived work-readiness, and facilitate personal development, and interpersonal skills. To ensure that systems related capabilities are well-developed, educators should regulate students’ academic workload while on placement, and consider including health-system-related aspects of professional practice such as business and practice management and clinical governance in the curriculum to provide students with the skills needed to foster readiness for transition into the health workforce.

Furthermore, considering the current interest in research on work-readiness among multi-disciplinary health professionals, longitudinal studies may make a worthwhile contribution to gain participants’ insights and reflections on practice after commencing work. This is because preparedness is a continuous non-linear process, which should be assessed as part of an integrated, continuous assessment model encompassing both training and practice [57].

### Strengths and Limitations

This study has several strengths that enhance its contribution to existing literature. Firstly, it gathered the perceptions of students across multiple health profession disciplines, increasing the generalisability of the findings. Secondly, the mixed-methods approach elucidated more in-depth information on student transition experiences by comparing and contrasting multiple datasets. Thirdly, the regional location of the university and its focus on regional and rural health provide insight into health professions students' readiness for practice in circumstances where professional collaboration and resources are limited. However, transferability of the study findings may be limited by the variation in the number of representative students from each health profession discipline. A much higher percentage of dentistry students participated in the study, potentially leading to an over-representation of their voices compared to the other disciplines. Additionally, participants were drawn from a single university and the study findings may not be applicable to settings where training requirements may differ. Finally, the students may have been less forthcoming in the focus groups due to the presence of their peers.

## Conclusions

Transitioning from university into the workforce may be stressful for health professions students due to uncertainties and expectations relating to system-based capabilities. Placement experiences, enriched with skills acquisition and a support system of supervisors, peers and family were seen as the biggest contributors to a smoother transition, making students more confident in their abilities to provide patient-centred care and carry out key tasks such as history taking and practising in a professional manner. Conversely, students felt less prepared to practice in private settings and felt limited by increased workload during placement. Recommendations to ease off the stress include better induction into placement sites and more support to foster system-related clinical governance training within the healthcare system. Engagement of educators in enhancing students’ familiarity with the clinical training environment will likely reduce uncertainty and facilitate a smoother transition into the health workforce. Longitudinal studies are recommended to provide better understanding of the forte of health educational programs as well as perceived gaps in junior health professionals’ readiness to practice and transition into the workforce.

## Data Availability

All data associated with this manuscript is published herein.

## Declarations

***Ethics***.

The JCU Human Ethics Review Committee granted ethics approval for the study (H7853). Written informed consent to participate was obtained from each participant.
